# Supplementary material for: Preliminary Assessment of the Possible Environmental Risks of Photopolymerizing Resin Particles Produced by Finishing Stereolithography 3D-Printed Objects, Employing Toxicity Test on Tropical House Crickets (Gryllodes sigillatus)
Source: Int J Mol Sci. 2025 Nov 21;26(23):11245. doi: 10.3390/ijms262311245 (PMC12691734; doi:10.3390/ijms262311245)
Supplement: Supplementary file 1 [file ijms-26-11245-s001.zip › S2 Detailed enzyme activity measurements script.pdf]

Detailed enzyme activity script

Total protein concentration

The total protein content was determined using the biuret method. For the assay, 5 µL of the sample, blank, and calibrator were pipetted in triplicate onto a 96-well plate, followed by the addition of 200 µL of the biuret reagent. The mixture was then shaken and incubated for 5 minutes at 37°C. After incubation, absorbance was measured twice at a wavelength of 540 nm (Fig. 1). The total protein content in the sample was calculated by substituting the measured absorbance values into the formula provided in the manufacturer's instructions:

$$\frac{\text{sample absorbance} - \text{blank absorbance}}{\text{calibrator absorbance} - \text{blank absorbance}} \times 7\ (\text{calibrator concentration})$$

Calibrator concentration provided by kit manufacturer – 7g/dL bovine albumin.

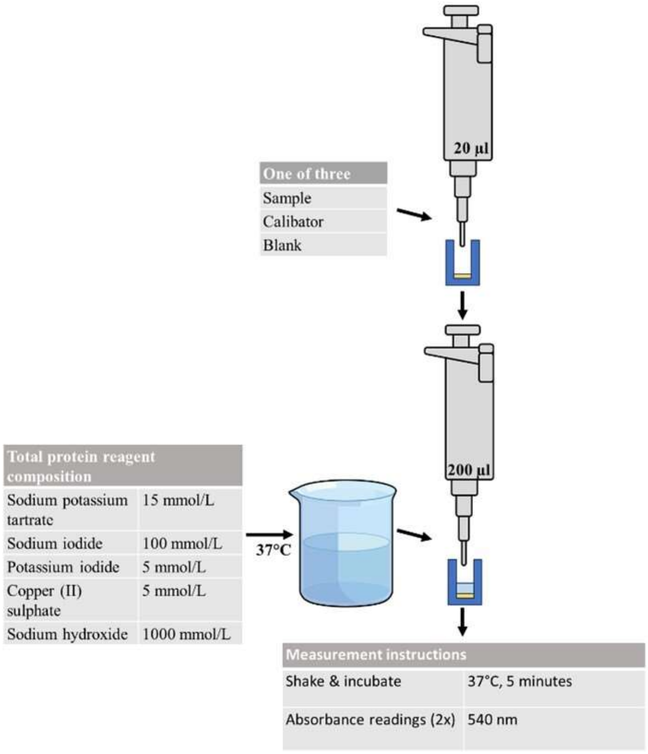

Figure 1. The scheme of total protein concentration measurement with adjusted volumes for 96-well plates. Reagent composition is shown on the left side.

ACP, ALT, AST activity

The activity of alanine aminotransferase (ALT) and aspartate aminotransferase (AST) was determined by measuring changes in dihydronicotinamide adenine dinucleotide (NADH) concentration. Acid phosphatase (ACP) activity was assessed on the formation of Azo dye. For the assay, 20 µL of the sample, blank, and calibrator were pipetted in triplicate onto a 96-well plate, followed by the addition of 200 µL of the reagent. The mixture was shaken and incubated at 37°C for 5 minutes (ACP) or 1 minute (ALT, AST). After incubation, the initial absorbance was measured at 405 nm for ACP and 340 nm for ALT and AST. The samples were then incubated for an additional 3 minutes, with absorbance measurements recorded at 1-minute intervals (Fig. 2).

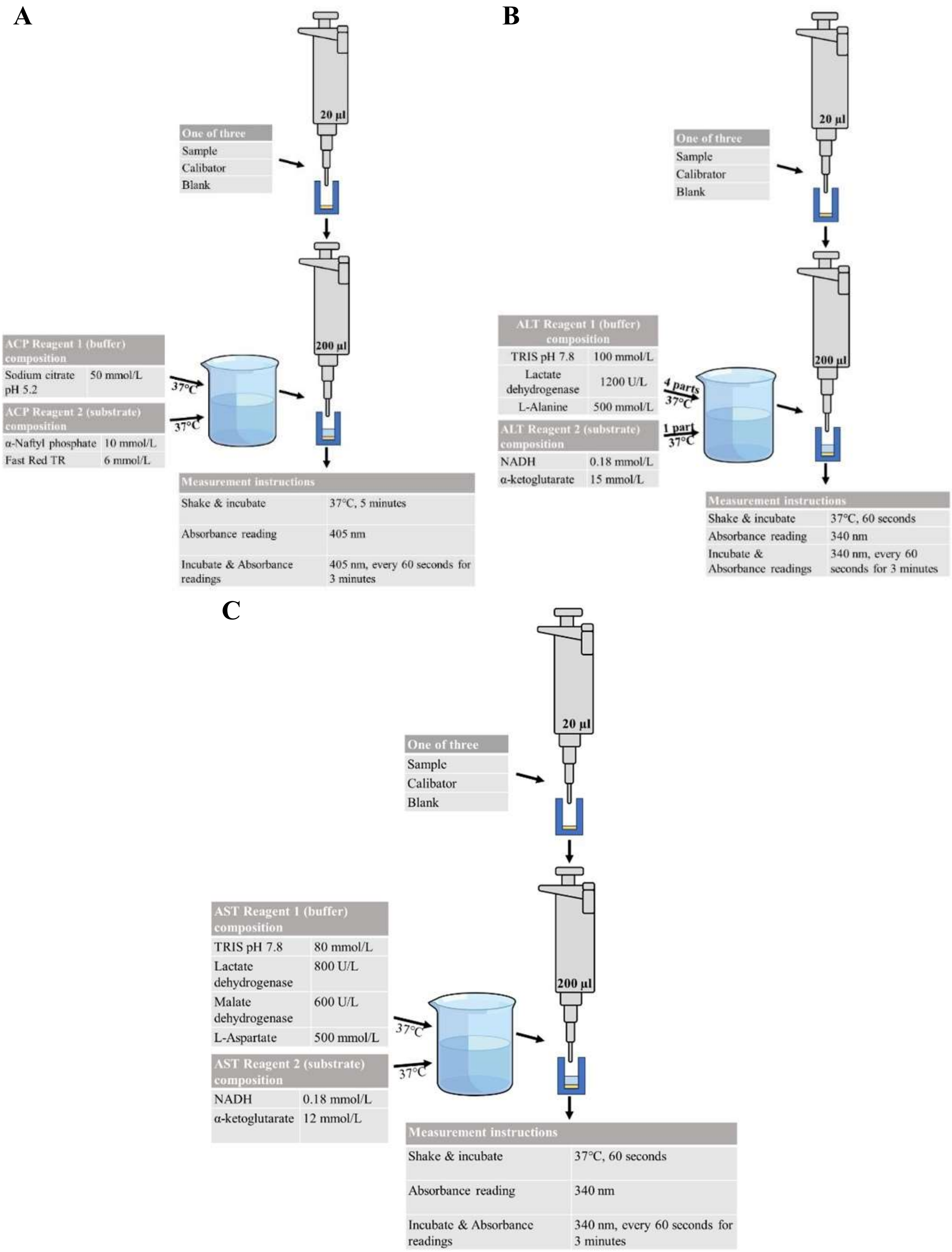

Figure 2. The schemes of acid phosphatase (ACP), alanine aminotransferase (ALT), aspartate aminotransferase (AST) [A-C, respectively] activity measurement with adjusted volumes for 96-well plates. Reagents compositions are shown on the left side.

ALP activity

The activity of alkaline phosphatase (ALP) was determined by measuring the change in p-nitrophenol concentration, which is generated through the hydrolysis of p-nitrophenyl phosphate by ALP. For the assay, 2 µL of the sample, blank, and calibrator were pipetted in triplicate onto a 96-well plate, followed by the addition of 120 µL of the reagent. The mixture was then shaken and incubated at 37°C for 1 minute. After incubation, the initial absorbance was measured at 405 nm, followed by an additional 3-minute incubation with absorbance readings recorded at 1-minute intervals (Fig. 3).

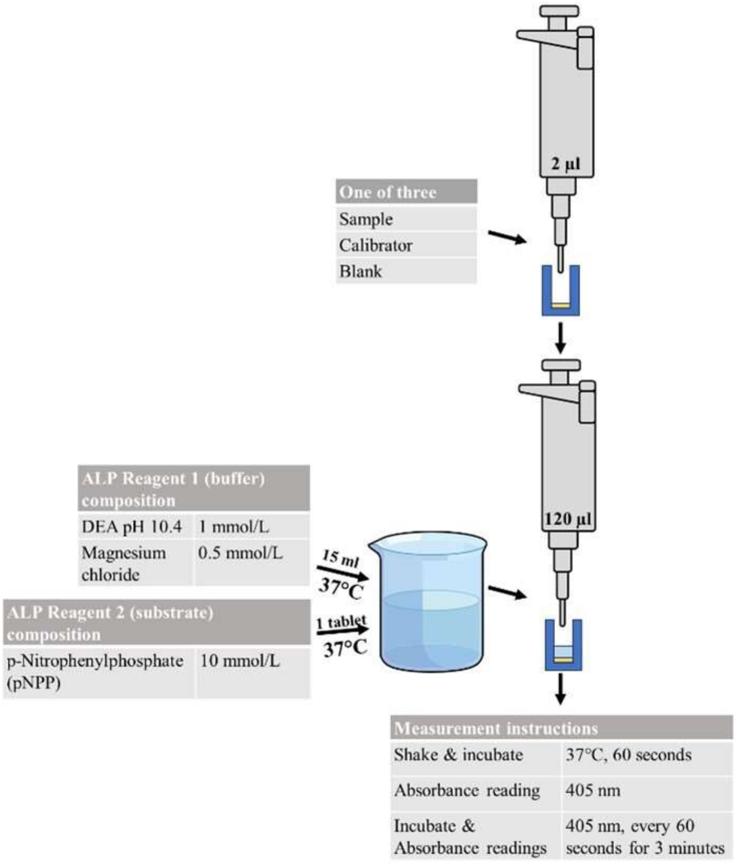

Figure 3. The scheme of alkaline phosphatase (ALP) activity measurement with adjusted volumes for 96-well plates. Reagent composition is shown on the left side.

GPX activity

The activity of glutathione peroxidase (GPX) was determined by measuring the change in nicotinamide adenine dinucleotide phosphate (NADPH) concentration. The method is based on that of Paglia and Valentine. For the assay, 4 µL of the sample, blank, and calibrator were pipetted in triplicate onto a 96-well plate, followed by the addition of 200 µL of reagent R1 and 8 µL of reagent R2. The mixture was then shaken and incubated at 37°C for 1 minute. After incubation, the initial absorbance was measured at 340 nm, followed by an additional 2-minute incubation with absorbance readings recorded at 1-minute intervals (Fig. 4).

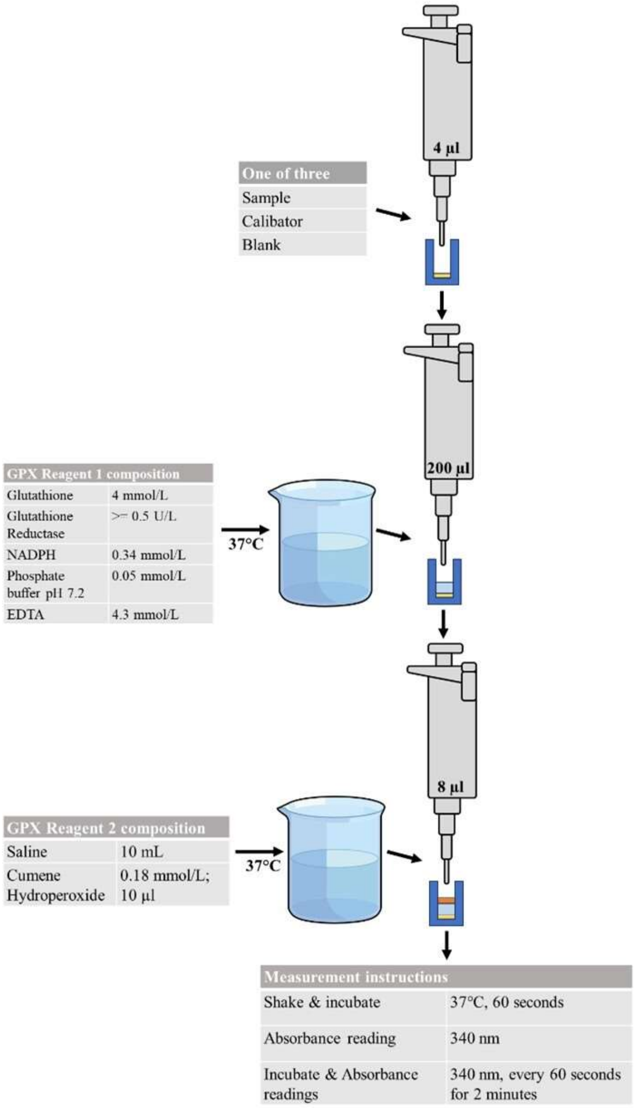

Figure 4. The scheme of glutathione peroxidase (GPX) activity measurement with adjusted volumes for 96-well plates. Reagent composition is shown on the left side.

SOD activity

The activity of superoxide dismutase (SOD) was determined by measuring the inhibition of reactive oxygen species through the change in the rate of formation of red formazan dyes. For the assay, 1 µL of the sample and 4 µL of diluent, which is 0.01M PBS, were pipetted in triplicate onto a 96-well plate. Additionally, 5 µL of blank and calibrators (5 dilutions of calibrator – 100% concentration, three 50% dilutions of the previous standard and one dilution 3 parts to 6 parts of the previous calibrator) — were pipetted in duplicate. This was followed by the addition of 200 µL of reagent R1 and 30 µL of reagent R2. The mixture was then shaken and incubated at 37°C for 30 seconds. After incubation, the initial absorbance was measured at 505 nm, followed by an additional 3-minute incubation with absorbance readings recorded at 1-minute intervals (Fig. 5).

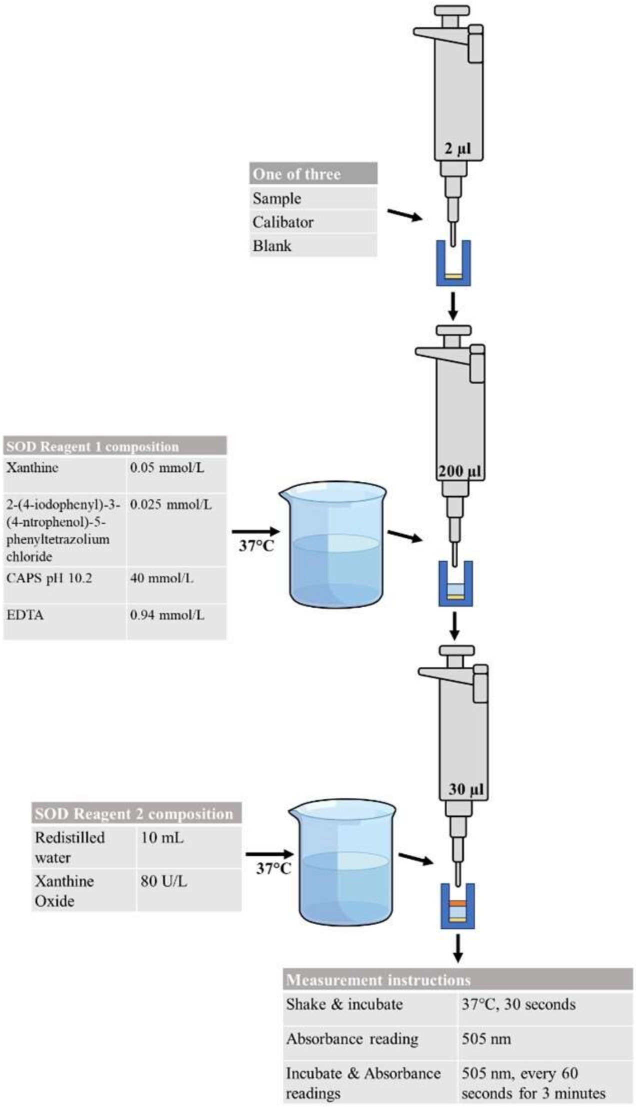

Figure 5. The scheme of superoxide dismutase (SOD) activity measurement with adjusted volumes for 96-well plates. Reagent composition is shown on the left side.

Lipase activity

Lipase activity was determined using a method based on the sequential hydrolysis of 1,2-O-dilauryl-rac-glycero-3-glutaric acid (6-methylresorufin) ester in the presence of colipase, deoxycholate, and calcium ions. For the assay, 2 µL of the sample was pipetted in triplicate onto a 96-well plate, followed by the addition of 200 µL of reagent R1 and 40 µL of reagent R2. The mixture was then shaken and incubated at 37°C for 60 seconds. After incubation, the initial absorbance was measured at 580 nm, followed by an additional 2-minute incubation with absorbance readings recorded at 1-minute intervals (Fig. 6). Lipase activity was calculated according to the formula provided by kit manufacturer.

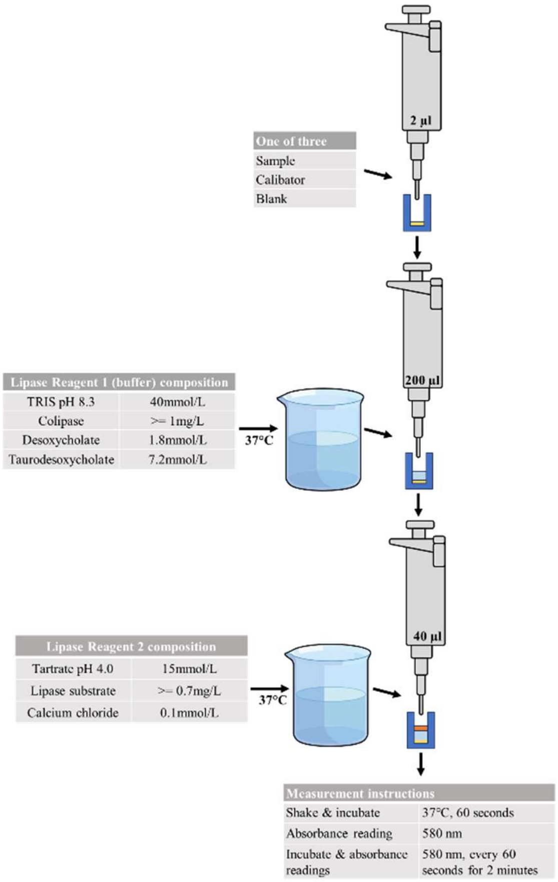

Figure 6. The scheme of lipase activity measurement with adjusted volumes for 96-well plates. Reagent composition is shown on the left side.

Amylase activity

Amylase activity, specifically α-amylase, was determined using a method based on the hydrolysis of 2-chloro-4-nitrophenyl-α-D-maltotrioside by monitoring the rate of 2-chloro-4-nitrophenol formation. For the assay, 5 µL of the sample was pipetted in triplicate onto a 96-well plate, followed by the addition of 250 µL of reagent. The mixture was then shaken and incubated at 37°C for 30 seconds. After incubation, the initial absorbance was measured at 405 nm, followed by an additional 3-minute incubation with absorbance readings recorded at 1-minute intervals (Fig. 7).

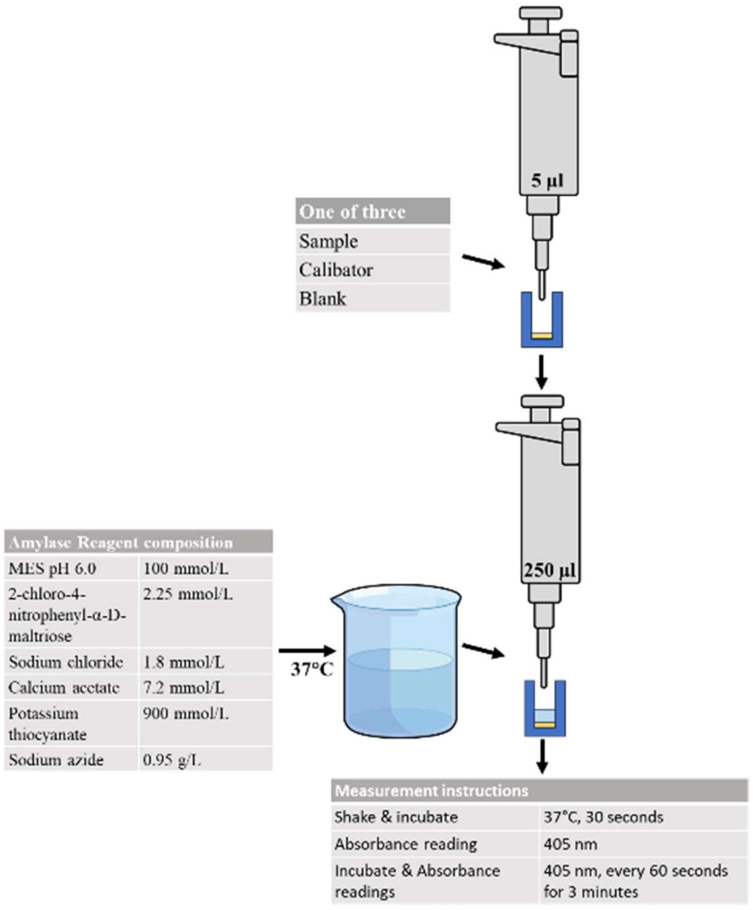

Figure 7. The scheme of amylase activity measurement with adjusted volumes for 96-well plates. Reagent composition is shown on the left side.
